# Patient- and provider-level risk factors associated with default from tuberculosis treatment, South Africa, 2002: a case-control study

**DOI:** 10.1186/1471-2458-12-56

**Published:** 2012-01-20

**Authors:** Alyssa Finlay, Joey Lancaster, Timothy H Holtz, Karin Weyer, Abe Miranda, Martie van der Walt

**Affiliations:** 1 Division of Tuberculosis Elimination, Centers for Disease Control and Prevention, 1600 Clifton Road NE, Atlanta, GA 30333, USA; 2 Tuberculosis Epidemiology and Intervention Research Unit, Medical Research Council, 1 Soutpansberg Road, Pretoria 0001, South Africa; 3 Stop TB Department, World Health Organization, 20 Avenue Appia, CH-1211 Geneva 27, Switzerland

**Keywords:** Tuberculosis, treatment default, non-adherence, South Africa

## Abstract

**Background:**

Persons who default from tuberculosis treatment are at risk for clinical deterioration and complications including worsening drug resistance and death. Our objective was to identify risk factors associated with tuberculosis (TB) treatment default in South Africa.

**Methods:**

We conducted a national retrospective case control study to identify factors associated with treatment default using program data from 2002 and a standardized patient questionnaire. We defined default as interrupting TB treatment for two or more consecutive months during treatment. Cases were a sample of registered TB patients receiving treatment under DOTS that defaulted from treatment. Controls were those who began therapy and were cured, completed or failed treatment. Two respective multivariable models were constructed, stratified by history of TB treatment (new and re-treatment patients), to identify independent risk factors associated with default.

**Results:**

The sample included 3165 TB patients from 8 provinces; 1164 were traceable and interviewed (232 cases and 932 controls). Significant risk factors associated with default among both groups included poor health care worker attitude (new: AOR 2.1, 95% CI 1.1-4.4; re-treatment: AOR 12, 95% CI 2.2-66.0) and changing residence during TB treatment (new: AOR 2.0, 95% CI 1.1-3.7; re-treatment: AOR 3.4, 95% CI 1.1-9.9). Among new patients, cases were more likely than controls to report having no formal education (AOR 2.3, 95% CI 1.2-4.2), feeling ashamed to have TB (AOR 2.0, 95% CI 1.3-3.0), not receiving adequate counseling about their treatment (AOR 1.9, 95% CI 1.2-2.8), drinking any alcohol during TB treatment (AOR 1.9, 95% CI 1.2-3.0), and seeing a traditional healer during TB treatment (AOR 1.9, 95% CI 1.1-3.4). Among re-treatment patients, risk factors included stopping TB treatment because they felt better (AOR 21, 95% CI 5.2-84), having a previous history of TB treatment default (AOR 6.4, 95% CI 2.9-14), and feeling that food provisions might have helped them finish treatment (AOR 5.0, 95% CI 1.3-19).

**Conclusions:**

Risk factors for default differ between new and re-treatment TB patients in South Africa. Addressing default in both populations with targeted interventions is critical to overall program success.

## Footnote page

All authors had access to this data and share responsibility for the decision to submit this manuscript for publication. All authors have provided a description of their contribution and have signed a statement granting exclusive license for publication to BMC Infectious Disease.

The views expressed within this paper are solely those of the authors and do not reflect those of the U.S. Public Health Service or the South African Medical Research Council. There are no financial, personal or professional interests that could be construed to influence the paper.

Trade names are used for identification only and do not represent endorsement by the Centers for Disease Control and Prevention or the U.S. Public Health Service.

One or more authors were employees of the US federal government when this work was conducted and prepared for publication; therefore, it is not protected by the Copyright Act, and copyright ownership cannot be transferred.

## Background

In 2002, South Africa ranked 9th worldwide in terms of total number of tuberculosis (TB) cases and 8th in terms of the highest TB incidence rates per capita. The incidence of all reported TB cases had increased 2.5 fold between 1992 (228/100,000) and 2002 (558/100,000) [1]. By 2001, TB was the leading reported cause of death by natural causes [2]. While case detection and implementation of DOTS increased substantially during this time frame, by 2002, treatment success rates remained low (65% for new cases and 53% for re-treatment cases), and default (12% for new cases and 17% for re-treatment cases) and death (7% for new cases and 9% for re-treatment cases) rates high [1].

Persons under treatment for TB who default from treatment are at risk for clinical deterioration and complications, can continue to be infectious to others, and are at risk of premature death from TB [3]. There are limited studies on the risk factors associated with TB treatment default in S. Africa under DOTS [4-7]. Having had a history of TB treatment has been associated with TB treatment default [8-10]. South Africa faces unique challenges with an unparalleled co-epidemic of TB and human immunodeficiency virus (HIV), in the setting of an overburdened health-service sector. There are complex economic and environmental conditions, including high levels of urban migration and unemployment, and unique socio-cultural risk factors that may have important influences on TB treatment adherence. The relative contribution of these factors to the problem of default from TB treatment is not known. We performed a retrospective case-control study to evaluate risk factors for default from TB treatment in 8 of 9 provinces in South Africa in 2002. Our primary objective was to identify patient-level and provider-level risk factors associated with default from TB treatment.

## Methods

We conducted a questionnaire-based, case-control study among adult persons ≥ 18 years old enrolled in treatment under DOTS at public health facilities in South Africa between January 1 and December 31, 2002. Eligible cases were defined as any new and re-treatment TB patient (with pulmonary or extrapulmonary TB) who were on treatment for at least 4 weeks, then defaulted from a six-month (new) or eight-month (re-treatment) TB regimen. New patients received a fixed dose combination Rifafour^® ^(isoniazid, rifampin, pyrazinamide, ethambutol), retreatment cases received Rifafour^® ^plus streptomycin injection.

We defined default as interrupting TB treatment for two or more consecutive months during treatment. Eligible controls were those persons diagnosed with TB who began therapy and were cured, completed or failed treatment. Persons were excluded if they were recorded to have died or transferred, were younger than 18 years, were known to have multidrug-resistant TB or were prisoners or wards of the state.

Interviewers were trained to review medical records available at the health facilities, and to trace and interview TB patients with written informed consent. Interviewers made every effort to locate patients using information available from the health center records or staff who knew the patients; they telephoned patients, located their homes if possible and made multiple visits if necessary. In the event a patient no longer lived at a specific address, they asked local neighbors and guides to help them trace the patients. To minimize bias, the interviewers did not know the patients and were not associated with the local health services. Data collected from the health facilities included demographic information, patient's address, treatment information including dates of TB registration, treatment initiation and completion and treatment outcome.

**Figure 1:**
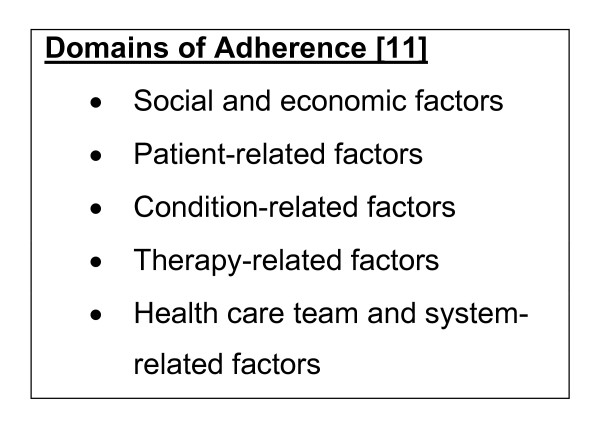
**Domains of adherence**.

A structured questionnaire was adapted from a questionnaire previously used in South Africa to study MDR TB adherence, with additional questions concerning the role of food and nutrition during TB treatment [6]. Questions covered five domains commonly cited regarding adherence to long-term therapies (Figure 1) [11]. The questionnaire was pre-tested and translated from English into 10 South African home languages (Xitsonga, Tshivenda, Siswati, Setswana, Sesotho, IsiZulu, IsiXhosa, IsiNdebele, Afrikaans) and back-translated into English to ensure the quality of translation. The questionnaire was a combination of multiple-choice, yes/no and open-ended questions on demographic, social, health service and treatment characteristics. We inquired about the provision of nutritional support and the role of enablers and incentives. Information on the HIV status of persons with TB was not included as HIV services were not integrated in the TB program in 2002.

Questionnaires were administered in the TB patient's home language using face-to-face interviews. Interviewers were trained to encourage defaulters to return to the health clinic to be evaluated for TB treatment. For patients who were found to have died, the outcome of death, date of death and cause, if available, was recorded. Patients were given a standard food parcel equivalent to approximately US$15 for participating in the study, offered after the informed consent and interview so as to not bias study participation or responses.

The sample was selected from facility-based national TB registers in 8 provinces and matched by quarter of enrolment and health facility at a ratio of 1 case: 2 controls. TB registers from Limpopo province were not available and thus excluded. Sample selection for each province was conducted by multi-stage sampling made up of urban and rural sub-samples that reflected the estimated demographic population proportions reported for 2001. For each province, we compiled a list of all urban and rural public clinics including number of new patients enrolled and number of defaulters in 2002. The health facility sub-samples were chosen by systematic sampling proportional to clinic enrolment size. TB registers were requested from selected health facilities and the pre-calculated number of cases and matched controls were selected randomly from the TB registers.

Based on historic program data on the proportion of patients from each province classified as re-treatment patients (8-27% depending on the province), we calculated a sample size large enough to show at least a 25% difference between cases and controls that would result in a statistically significant odds ratio (OR) of > 2.9 with a 95% confidence interval (CI) and a power of 80%, for each province. In anticipation of difficulties associated with finding defaulters, the overall sample size was increased by 75%. Based on these calculations, the targeted sample size for the 8 provinces was 1055 cases and 2110 controls.

Data were entered into an EpiData v.3.1 database and analyzed with STATA 8 [12,13]. Data were validated by checking consistency values, evaluating range values, and performing uniqueness checks. We analyzed differences in proportions using the Mantel-Haenszel chi-square statistic (*χ*^2^) or Fisher's exact test where appropriate. For data not normally distributed, we compared differences in medians using the Wilcoxon rank-sum test. We performed multivariable logistic regression analysis with forward selection. We evaluated all variables in the bivariate analysis that were significant at *P *< 0.20 in the multivariable analysis using logistic regression for the final models. For all statistical tests, we considered a *P *value of < 0.05 as statistically significant. All pair wise interactions of the explanatory variables that were epidemiologically relevant in the model were considered.

### Ethics statement

This study was approved by the Ethics Committee of the South African Medical Research Council and the respective provincial research committees. This study was also approved as human subjects' research by the Institutional Review Board at the US Centers for Disease Control and Prevention in Atlanta, GA, USA. Voluntary written informed consent was obtained from all participants included in the study.

## Results

**Figure 2:**
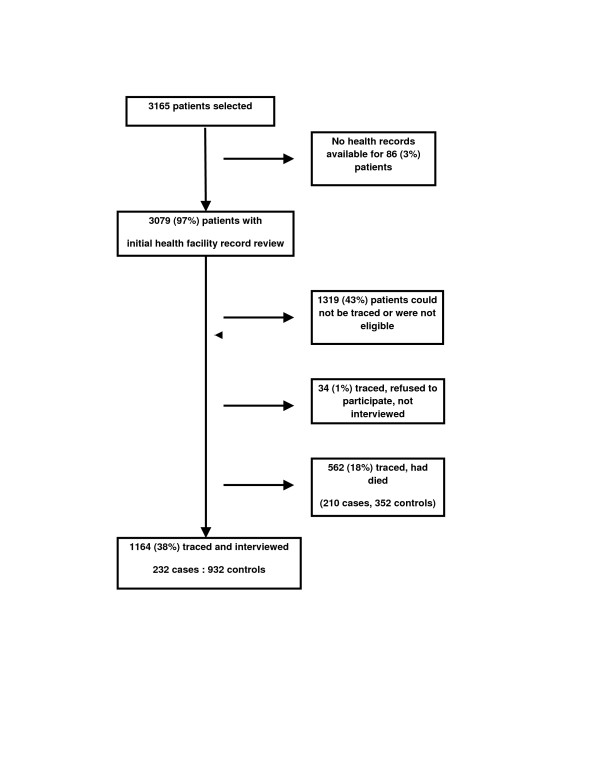
**Interview rate**.

We collected data between July 2004 and August 2005. Of 3165 patients sampled, health records were available for 3079 (97%). Of these, we successfully traced and interviewed 1164 (232 cases and 932 controls, 26% and 43% respectively) (Figure 2). Few patients refused to be interviewed (34 patients, 1%). An additional 1319/3079 (43%) patients could not be traced, due to incorrect addresses listed in health facility records or were not eligible. A large proportion (18%) of patients were found to have died, including 210/900 (23%) cases and 352/2179 (16%) controls (*P *< 0.001). The date of death was known for 115/200 (55%) cases. Of these, 33 (22%) were reported to have died within 2 months after stopping TB treatment before completion (no data on when during treatment). These patients were probably misclassified as defaulters, and were likely deaths.

Limited clinical data were available from patients who were not interviewed. Cases interviewed vs. not interviewed were more likely to be younger, to have attended a rural clinic, to be a re-treatment patient, and to default later in the treatment course. Controls interviewed vs. not interviewed were more likely to have attended a rural clinic, to be a re-treatment patient and less likely to have been admitted to an inpatient healthcare facility for TB treatment. Patients who were not interviewed were excluded from further analysis.

We explored factors associated with default, stratified among new and re-treatment patients. Among the 1164 patients interviewed, 2/1164 had an unknown patient category and were excluded from the analysis. Tables 1, 2 and 3 summarize the main characteristics from the health facility record review and interviews with 1162 patients (231 cases and 931 controls) who had a known patient category, were successfully traced, and consented to participate.

**Table 1:** Sex and age distribution of 231 cases and 931 controls with TB, stratified by patient category, South Africa, 2002*

		New Patients (n = 926)			Re-treatment Patients (n = 236)		
**Factor**		**Cases (n = 160)**	**Controls (n = 766)**	**Stratum specific OR** **(95% CI)**	**Cases (n = 71)**	**Controls(n = 165)**	**Stratum specific OR** **95% CI**

Sex	Male	100/160	398/766	1.5 (1.1-2.2)	54/71	118/165	1.3 (0.6-2.3)

	Female	60/160	368/766	1.0	17/71	47/165	1.0

Median age		30	34	p = 0.001	33	39	p = 0.003

The patient category was not documented for one case patient

*One person's age was unknown

**Table 2:** Social and economic characteristics of cases and controls with TB, stratified by patient category, South Africa, 2002

		New TB Patient (N = 926)					Re-treatment TB Patient (N = 236)					Combined	
		
		Cases (160)		Controls (766)		OR (95% CI)	Cases (71)		Controls (165)		OR (95% CI)	OR*(95% CI)	AOR† (95% CI)
**Characteristic**		**n/N**	**%**	**n/N**	**%**		**n/N**	**%**	**n/N**	**%**			

Country of birth	RSA	159/160	99	745/766	97	4.5 (0.7-186)	71/71	10	163/164	99	n/a	5.6 (0.9-231)	4.9 (0.7-36)
	
	Outside RSA	1/160	1	21/766	3		0/71	0	1/164	1			

Practices religion	Yes	148/160	93	687/758	91	1.3 (0.7-9)	63/71	89	151/162	93	0.6 (0.2-1.7)	1.0 (0.6-1.8)	1.0 (0.6-1.7)
	
	No	12/160	7	71/758	8		8/71	11	11/162	7			

Race	Black	129/159	81	661/762	86	0.7 (0.4-1.1)	57/71	80	129/164	79	1.1 (0.5-2.4)	0.7 (0.5-1.1)	0.8 (0.5-1.1)
	
	Other	30/159	19	101/762	14		14/71	20	35/164	21			

Marital status	Married or permanent relationship	50/158	32	266/763	35	0.9 (0.6-1.3)	23/71	32	61/164	37	0.8 (0.4-1.5)	0.9 (0.6-1.2)	0.8 (0.6-1.2)
	
	Single	108/158	68	497/763	65		48/71	68	103/164	63			

Biological children	No children	31/157	20	147/761	19	1.0 (0.6-1.6)	21/70	30	31/164	19	1.8 (0.9-3.7)	1.2 (0.9-1.8)	1.2 (0.9-1.7)
	
	≥ 1 child	126/157	80	614/761	81		49/70	70	133/164	81			

Formal Education	None	22/160	14	61/762	8	1.8 (1.0-3.1)	5/70	7	17/164	10	0.7 (0.2-2.0)	1.4 (0.9-2.3)	1.4 (0.9-2.3)
	
	Some	138/160	86	701/762	92		65/70	93	147/164	90			

Owns a house	Yes	81/157	52	386/759	51	1.0 (0.7-1.5)	37/71	52	89/164	54	0.9 (0.5-1.6)	1.0 (0.7-1.5)	1.0 (0.7-1.3)
	
	No	76/157	48	373/759	49		34/71	48	75/164	46			

Owns cattle	Yes	10/157	6	31/755	4	1.6 (0.7-3.4)	2/71	3	9/164	3	0.5 (0.05-2.5)	1.2 (0.6-2.4)	1.2 (0.6-2.3)
	
	No	147/157	94	724/755	96		9/71	97	155/164	97			

Owns radio	Yes	111/158	70	500/759	66	1.2 (0.8-1.8)	45/71	63	115/164	70	0.7 (0.4-1.4)	1.1 (0.8-1.5)	1.1 (0.7-1.5)
	
	No	47/158	30	259/759	34		26/71	27	49/164	30			

Changed residence during TB treatment	Yes	22/158	14	49/756	6	2.3 (1.3-4.1)	16/71	23	12/165	7	3.7 (1.5-9.1)	2.8 (1.8-4.2)	2.7 (1.7-4.2)
	
	No	136/158	86	707/756	94		55/71	77	153/165	93			

Employed	Yes	81/157	52	386/759	51	1.0 (0.7-1.5)	37/71	52	89/164	54	0.9 (0.5-1.6)	1.0 (0.7-1.4)	1.0 (0.7-1.3)
	
	No	76/157	48	373/759	49		34/71	48	75/164	46			

Type of employment	Laborer	29/83	35	88/370	24	1.7 (1.0-2.9)	13/38	34	19/81	23	1.7 (0.7-1.3)	1.7 (0.6-4.3)	1.7 (1.1-2.6)
	
	Other	54/83	65	282/370	76		25/38	66	62/81	77			

Missed treatment due to employment	Yes	39/85	46	16/381	4	19 (9.6-40)	14/40	35	4/80	5	10 (2.8-45)	16 (9-31)	16 (9-29)
	
	No	46/85	54	365/381	96		26/40	65	76/80	95			

In prison during treatment	Yes	7/156	5	25/748	3	1.4 (0.5-3.3)	6/71	8	11/165	7	1.3 (0.4-4.0)	1.5 (0.7-2.9)	1.3 (0.7-2.6)
	
	No	149/156	95	723/748	97		65/71	92	154/165	93			

*Combined bivariate odds ratio

† Odds ratio adjusted for patient category

**Table 3:** Patient, condition and therapy-related factors of cases and controls with TB, stratified by patient category, South Africa, 2002

		New TB Patient (N = 926)					Re-treatment TB Patient (N = 236)					Combined	
		
		Cases (160)		Controls (766)		OR(95% CI)	Cases (71)		Controls (165)		OR (95% CI)	OR*(95% CI)	AOR† (95% CI)
**Characteristic**		**n/N**	**%**	**n/N**	**%**		**n/N**	**%**	**n/N**	**%**			

Felt ashamed about TB	Yes	67/155	43	181/753	24	2.4 (1.6-3.5)	31/71	44	48/164	30	1.9 (1.0-3.5)	2.3 (1.7-3.1)	2.2 (1.6-3.0)
	
	No	88/155	67	572/753	76		40/71	56	116/164	70			

Felt supported by family	Yes	116/153	76	651/736	88	0.4 (0.2-0.6)	54/67	80	140/160	88	0.6 (0.3-1.4)	0.5 (0.3-0.7)	0.5 (0.3-0.7)
	
	No	37/153	24	85/736	12		13/67	20	20/160	22			

Knew treatment duration was ≥ 6 months	Yes	108/149	72	623/741	84	0.9 (0.4-2.0)	55/69	80	133/163	82	0.5 (0.3-0.8)	0.6 (0.4-0.8)	0.6 (0.4-0.8)
	
	No	41/149	28	118/741	16		14/69	20	30/163	18			

Felt they might die from TB	Yes	96/154	62	511/749	68	0.8 (0.5-1.1)	41/65	63	118/159	74	0.6 (0.3-1.2)	0.7 (0.5-1.0)	0.7 (0.5-1.0)
	
	No	58/154	38	238/749	32		24/65	37	41/159	26			

Thought they would finish taking treatment	Yes	112/143	78	573/734	78	1.0 (0.6-1.6)	52/63	63	130/152	74	0.8 (0.3-2.0)	1.0 (0.7-1.5)	1.0 (0.7-1.4)
	
	No	31/143	22	161/734	22		11/63	17	22/152				

Felt that it is possible to cure TB	Yes	119/123	97	729/738	99	0.4 (0.1-1.7)	58/62	94	149/153	97	0.4 (0.1-2.2)	0.3 (0.1-0.9)	0.4 (0.2-0.9)
	
	No	4/123	3	9/738	1		4/62	6	5/153	3			

Saw a traditional healer during TB treatment	Yes	30/159	19	52/757	7	3.2 (1.8-5.3)	9/69	13	12/165	7	1.9 (0.7-5.2)	2.8 (1.7-4.3)	2.8 (1.8-4.3)
	
	No	129/159	18	705/757	93		60/69	87	153/165	93			

Type of TB	Pulmonary	150/157	96	732/749	98	0.9 (0.2-1.4)	63/69	99	157/161	98	0.3 (0.05-1.2)	0.4 (0.2-0.9)	0.4 (0.2-0.8)
	
	Extra Pulmonary	7/157	4	17/749	2		6/69	1	5/161	2			

Alcohol use	Yes	51/154	33	157/753	21	1.9 (1.3-2.8)	27/70	39	46/163	28	1.6 (0.8-2.3)	1.9 (1.3-2.6)	1.8 (1.3-2.5)
	
	No	103/154	77	596/753	79		43/70	61	117/163	72			

Marijuana/mandrax use	Yes	6/159	4	29/756	4	1.0 (0.3-2.5)	8/71	11	9/165	5	2.2 (0.7-6.7)	1.5 (0.7-2.9)	1.4 (0.7-2.6)
	
	No	153/159	96	727/756	96		63/71	89	156/165	95			

History of default	No	n/a	n/a	n/a	n/a	n/a	26/165	16	38/71	53	6.2 (3.1-12)	n/a	n/a
	
	Yes	n/a	n/a	n/a	n/a	n/a	139/165	84	33/71	47			

Hospitalized for TB treatment	Yes	54/155	35	287/758	38	0.9 (0.6-1.1)	25/70	36	66/163	40	0.8 (0.4-1.5)	0.9 (0.6-1.2)	0.9 (0.6-1.2)
	
	No	101/155	65	471/758	62		45/70	64	97/163	60			

Felt better with treatment	No	32/150	21	15/754	2	13.3(7.0-27)	18/67	27	5/162	3	11.5 (3.9-41)	13.3 (7.5-24)	12.7 (7.3-22)
	
	Yes	118/150	79	739/754	98		49/67	73	157/162	97			

Treatment side effects	Yes	73/138	53	297/679	44	1.4 (1.0-2.2)	39/60	65	79/152	52	1.7 (0.9-7.8)	1.6 (1.2-2.2)	1.5 (1.1-2.1)
	
	No	65/138	47	382/679	56		21/60	35	73/152	48			

Spent ≥ 1 day without food during treatment	Yes	28/155	18	98/759	13	1.5 (0.9-2.4)	16/70	23	30/163	18	1.3 (0.6-2.7)	1.5 (1.0-2.2)	1.4 (0.9-2.1)
	
	No	127/155	82	661/759	87		54/70	77	133/163	82			

Took TB treatment on an empty stomach	Yes	49/142	35	183/725	25	1.6 (1.0-2.3)	24/62	39	43/160	27	1.7 (0.9-3.3)	1.6 (1.2-2.3)	1.6 (1.2-2.2)
	
	No	93/142	65	542/729	75		38/62	61	117/160	73			

*Combined bivariate odds ratio

† Odds ratio adjusted by patient category

### Social and economic characteristics

Table 1 shows the age distribution and sex of patients, stratified by patient category, new and re-treatment patients. Among new patients, cases were more likely to be male (OR 1.5, 95% CI 1.1-2.2). Among both new and retreatment patients, the median age of cases was younger than controls. Most patients were born in South Africa, single, black African, reported practicing religion, and had one or more children (Table 2). Unemployment was common and similar between groups (47% of cases and 52% of controls). Among employed patients, default was associated with patients missing treatment due to employment. Reasons cited by patients included that they were too busy and did not have enough time, work was too far from the TB clinic, employer did not allow them to get TB treatment and some patients did not want other co-workers to know they had TB.

Among new TB patients, cases were more likely than controls to be male, to lack formal education, to have changed residence during TB treatment, to be a laborer, and to have missed treatment due to employment. Among re-treatment TB patients, cases were more likely than controls to have changed residence during TB treatment and to have missed treatment due to employment. New and re-treatment cases and controls were similar in terms of feeling ashamed, seeing a traditional healer, alcohol use, and experiencing side effects (Table 3).

### Patient-related factors

Most patients reported having told someone about their TB diagnosis, and these findings were similar between cases and controls. However, among both new and re-treatment groups, cases were more likely than controls to report feeling shame or embarrassment about having TB (Table 3). Defaulters were also more likely than controls to report that they did not feel better with treatment. Among new TB patients, cases were less likely than controls to have felt supported by their family during TB treatment. Reported causes of TB were similar between groups; although, some incorrect causes were commonly cited by both cases and controls including "witchcraft" and "taboo". The majority of cases and controls reported that they originally thought they would have finished taking treatment, felt that TB was curable, and that they could die from TB if not treated.

### Therapy and condition-related factors

New and re-treatment cases and controls were similar in terms of their type of TB (pulmonary vs. extrapulmonary), hospitalization for TB treatment, history of previous treatment failure and having treatment side effects. Among new TB patients, cases were more likely than controls to have taken TB treatment on an empty stomach, to have seen a traditional healer while on treatment for TB and to use alcohol during treatment. Among re-treatment patients, cases were more likely than controls to have a history of previous default. Among both groups, cases were more likely than controls to report that treatment did not make them feel better.

### Health care team and system-related factors

Cases and controls were similar in terms of type and location of facility they attended for treatment, their reported access to the clinic, the number of clinics attended, and whether they took their treatment under direct observation. Half of cases and controls reported taking treatment without direct supervision despite the fact that they were all enrolled in DOTS. However, the two groups reported marked differences in opinion about experiences with health services and the health staff during their TB treatment. Cases were more likely than controls to report that clinic hours were inconvenient, that the health care worker (HCW) had a negative attitude towards them, did not treat them with respect, that they did not trust the HCW and that they missed treatment because of poor HCW attitude (Table 4). Cases were also more likely than controls to report that they had not received enough education about TB at the beginning of treatment, that they were not told why treatment would take 6 or more months and lacked counsel and information about TB treatment in general. Among retreatment patients, cases were more likely than controls to report the food from the clinic would help them finish taking their TB treatment.

**Table 4:** Health care team and system related factors, cases and controls with TB, stratified by patient category, South Africa, 2002

		New TB Patient (N = 926)					Re-treatment TB Patient (N = 236)					Combined	
		
		Cases (160)		Controls (766)		OR(95% CI)	Cases (71)		Controls (165)		OR(95% CI)	OR*(95% CI)	AOR† (95% CI)
**Factor**		**n/N**	**%**	**n/N**	**%**		**n/N**	**%**	**n/N**	**%**			

Clinic hours convenient	No	32/147	22	60/747	8	3.2 (1.9-5.2)	12/64	19	8/160	5	4.4 (1.5-13)	3.2 (2.1-5.0)	3.4 (2.2-5.2)
	
	Yes	115/147	78	687/747	92		52/64	81	152/160	95			

Time spent waiting at clinic	≤ 1 hour	15/146	10	74/709	10	1.0 (0.5-1.8)	9/66	14	13/154	8	1.7 (0.6-4.6)	1.1 (0.7-1.9)	1.1 (0.7-1.9)
	
	> 1 hour	131/146	90	635/709	90		57/66	86	141/154	92			

Did HCW treat you with respect?	No	11/158	7	19/761	3	2.9 (1.2-6.6)	10/71	14	3/165	2	8.9 (2.1-51)	4.1 (2.1-8.0)	4.1 (2.2-7.8)
	
	Yes	147/158	93	742/761	97		61/71	86	162/165	98			

HCW attitude	Poor	17/160	11	30/763	4	2.9 (1.5-5.6)	12/71	17	5/164	3	6.5 (2.0-24)	3.6 (2.1-6.3)	3.7 (2.2-6.2)
	
	Good	143/160	89	733/763	96		59/71	83	159/164	97			

Missed treatment due to HCW attitude	Yes	15/155	10	19/763	25	4.2 (1.9-8.9)	9/69	13	1/162	1	24.2(3.1-1064)	5.4 (2.8-11)	5.8 (3.0-11)
	
	No	140/155	90	744/763	75		60/69	87	161/162	99			

Trusted HCW	No	9/156	6	14/749	2	3.2 (1.2-8.1)	5/66	8	4/163	25	3.3 (0.7-17)	3.3 (1.5-7.2)	3.2 (1.6-6.7)
	
	Yes	147/156	94	735/749	98		61/66	92	159/163	75			

Told why TB treatment must be taken ≥ 6 months	No	73/152	48	225/746	30	2.1 (1.5-3.1)	31/70	44	47/162	29	1.9 (1.0-3.6)	2.0 (1.5-2.8)	2.1 (1.5-3.1)
	
	Yes	79/152	52	521/746	70		39/70	56	115/162	71			

Given counsel or information on TB treatment	No	53/155	34	163/756	22	1.9 (1.3-2.8)	27/69	39	40/164	24	2.0 (1.0-3.8)	1.9 (1.4-2.7)	1.9 (1.4-2.6)
	
	Yes	102/155	26	593/756	78		42/69	61	124/164	76			

Received enough education about TB at beginning of treatment	No	17/98	17	44/592	7	2.6 (1.3-4.9)	10/39	26	3/121	2	13.6 (3.1-80)	3.5 (2.0-5.9)	3.6 (2.1-6.2)
	
	Yes	81/98	83	548/592	93		29/39	74	118/121	88			

Took pills without direct supervision (DOT)	Yes	77/148	52	400/741	54	0.9 (0.6-1.3)	32/70	46	55/160	34	1.6 (0.8-3.0)	1.0 (0.7-1.3)	1.1 (0.8-1.4)
	
	No	71/148	48	341/741	46		38/70	54	105/160	66			

Agreed that if food was provided by clinic, adherence to treatment would have been better	Yes	114/139	82	553/688	80	1.1 (0.6-1.9)	63/68	93	114/151	76	4.1 (1.4-14)	1.5 (1.0-2.4)	1.5 (1.0-2.3)
	
	No	25/139	18	135/688	20		5/68	7	37/151	24			

*Combined bivariate odds ratio

† Odds ratio adjusted by patient category

Two multivariable models were created, one for new patients and one for re-treatment patients (Table 5). Two risk factors were independently associated with default among both new and re-treatment patients: perception of poor HCW attitude and changing residence during TB treatment. Among new patients, additional risk factors associated with default were: not receiving adequate counseling or information during TB treatment (Adjusted OR 1.9, 95% CI 1.2-2.8), having no formal education (AOR 2.3, 95% CI 1.2-4.2), drinking any alcohol during TB treatment (AOR 1.9, 95% CI 1.2-3.0), feeling ashamed to have TB (AOR 2.0, 95% CI 1.3-3.0), and seeing a traditional healer during TB treatment (AOR 1.9, 95% CI 1.1-3.4). Among re-treatment patients risk factors associated with default included: a history of previous TB treatment default (AOR 6.4, 95% CI 2.9-14.0), not feeling better with TB treatment (AOR 21, 95% CI 5.2-84), and agreement with the statement "food given to me by the nurses at the clinic would better help me finish my TB treatment" (AOR 5.0, 95% CI 1.3-19).

**Table 5:** Multivariable logistic regression analysis for TB treatment default, stratified by patient category (new, re-treatment), South Africa, 2002*

	New Patients (848)		Re-treatment Patients (211)	
**Risk Factor**	**Adjusted OR**	**95% CI**	**Adjusted OR**	**95% CI**

Poor HCW attitude	2.1	1.1-4.4	12	2.2-66

Changing residence during TB treatment	2.0	1.1-3.7	3.4	1.1-9.9

Did not receive adequate counseling or information	1.9	1.2-2.8		

No formal education	2.3	1.2-4.2		

Drank any alcohol during treatment	1.9	1.2-3.0		

Felt ashamed to have TB	2.0	1.3-3.0		

Saw a traditional healer for TB	1.9	1.1-3.4		

History of previous TB default			6.4	2.9-14

Felt better with treatment			21	5.2-84

Agreed that if food was provided by clinic, adherence to treatment would have been better			5.0	1.3-19

*Models adjusted for age and sex; other variables considered but not included in the model: race, marital status, employment status, rural or urban setting

## Discussion

TB treatment interruption often leads to poor final treatment outcomes and drug-resistance, which has been a challenge for the Department of Health in South Africa for more than two decades. In our case control study of TB patients treated in 2002, only 26% of cases and 43% of controls could be traced and is an important limitation of the study. Nevertheless, we identified several potentially modifiable risk factors associated with treatment default. Notably, we found an association between default and poor perceived relations between the HCW and patient. The importance of this relationship and its influence on adherence to treatment is well established. A few studies have reported specifically on patient's satisfaction with health worker attitude and service provision and risk of default [6,11,14-16]. Vijay et. al found similar poor provider patient relationship factors associated with default among new smear positive TB patients [16]. Some studies have reported communication barriers or poor communication between patients and providers as being linked to poor adherence [15,17,18]. Perceived communication quality or lack thereof may reflect the status of the patient provider relationship and influence patient behavior [18,19]. Other studies have reported associations between TB treatment default and a poor level of knowledge of TB or low patient satisfaction regarding information provided concerning their illness [17-20]. Patient education materials should be appropriately tailored to education-level and a non-literate audience as needed.

Ensuring continuity of TB services among patients who are mobile is challenging. Our findings that patient mobility (changing residence) was associated with default underscores the need for improved communication and coordination between the patient and health services. Holtz, et. al., also found that changing residence was independently associated with MDR TB treatment default among MDR TB patients in South Africa [6]. The association between migration and default has been cited in other settings [9,20]. Patients require education about the ability to transfer care and TB programs should to be designed to closely follow-up and effectively refer patients to alternate DOTS treatment sites in a timely manner when required.

Among new TB patients, feeling shame was associated with default. Qualitative and quantitative studies among patients and providers have identified TB stigma as a likely barrier to adherence [17,21-23]. The growth of the HIV and TB co-epidemic and the well known relationship between the two diseases may have intensified the problem of TB stigma in South Africa [24]. Successful interventions such as community education and integrating community involvement in TB control may help to reduce social barriers to treatment and stigma [25,26].

Alcohol use was also associated with default among new TB patients. Alcohol use or alcohol abuse has been frequently reported as a risk factor for default [8,9,16,27-30]. While it can be difficult for patients to change this behavior, effective primary care behavior interventions to reduce patient alcohol use have been demonstrated [31,32].

The use of traditional healers is common in South Africa. Among new patients, use of traditional healers during TB treatment was associated with default. Several reports have described how seeking care from traditional healers can delay prompt diagnosis and treatment for TB and can negatively influence morbidity and mortality from TB [33-35]. Some TB case finding and treatment models have successfully partnered with traditional healers to implement community-based DOTS with good outcomes [36,37]. Every effort should be made to expand these successful projects, educate and collaborate with traditional healers and involve them in TB control.

An association between a previous history of default and subsequent default was found in our study and has been reported in several other settings [38-41]. Efforts to prevent first-time default among new patients, and methods to rapidly identify and intervene with previous defaulters who are at high risk are needed. As "felt better with treatment" was also associated with default among re-treatment patients, every effort should be made to monitor response to therapy and manage potential concurrent illnesses in an integrated and timely fashion.

It is concerning that half of all patients reported that they were not supervised when they took their TB treatment. Implementation of DOT services has been a challenge in South Africa [42]. Ntshanga, et. al., conducted an evaluation of the DOTS program in crisis districts in KwaZulu-Natal and showed poor implementation of DOT where low coverage, low quality and high caseloads were associated with poorer outcomes [43].

The general health care crisis, shortage of health workers and increased health care burden (largely from the HIV epidemic) in South Africa has led to compromises in quality of care, inadequate service delivery and is reflected in our study findings. The variety of factors associated with TB treatment default identified, touching upon each of the classic adherence domains, suggests a multi-pronged approach is needed.

Successful small scale comprehensive treatment models to improve TB treatment adherence have been evaluated and documented [43-47]. Key elements of these models include: (1) enhanced training for HCWs in communication and counseling skills, emphasizing patient-centered approaches; (2) development and use of effective and appropriate patient education materials, with an emphasis on patient adherence and self-monitoring tools; (3) close clinical follow-up; (4) effective incentives; (5) social assistance programs; (6) improved community participation, providing flexibility in DOT provider and decentralizing treatment through community based DOTS; (7) integrated TB and HIV care; (8) enhanced program supervision and management; and (9) adequate financial and human resources.

There were several limitations of this study. The low response rate limits power to draw generalized conclusions about South Africa. Other retrospective case control studies have reported similar difficulties in locating patients after they have left care at the TB program, especially defaulters [6,15-17,48-50]. The retrospective nature of the study and self-reported data collected from patients is subject to recall bias which may reduce the risk estimate. Cases interviewed were more likely to default later in the treatment course than those not interviewed. Because there is an unknown temporal association between the risk factors identified and treatment default, this limits our ability to potentially identify time-points in case management at which different risk factors for non-adherence are more important and where specific types of adherence strategies may have an increased impact.

Lastly, there are inherent difficulties in collecting subjective data between such disparate subjects as social factors, economic factors, health care team characteristics, and patient-level factors all reported through one mechanism, the patient self-report. The self-report refers to experience, rather than to reality. It is the experience (of reality and not reality itself) that informs future decisions by people about adherence and retention. Hence if we are interested in understanding the relationship between satisfaction and adherence, we need to know patient subjective experiences. The same applies to experiences that are quantifiable and can actually be verified from institutional data, such as the number of doctors seen, waiting times, etc. Also here the subjective experience is the overarching factor when it comes to making decisions by patients. Some of these factors we measured have direct impact on health care services, and others have indirect but equally large impacts on services. Unfortunately it was also not possible to include the provider perspective (health care workers and treatment supporters) on default and non-adherence in this study.

## Conclusion

Standard 9 of the International Standards of Tuberculosis Care includes creating a treatment environment to promote adherence and monitoring results using a patient centered approach and fostering respect between the patient and provider [51]. There are now several small scale models incorporating patient-centered approaches showing success at achieving good TB treatment outcomes in local settings in South Africa and other countries. Adequate resources, good program management, supervision and ongoing evaluation are urgently needed to ensure successful scale up and sustainability of these programs. While South Africa faces challenges of an overburdened health system amidst the escalating TB/HIV co-epidemic, the country has made policy changes and begun implementing innovative ways to reduce staff shortages, improve staff motivation, develop new approaches to clinical practice and education [52]. These promising changes provide a fertile ground on which to institute interventions to reduce default from TB treatment.

## Abbreviations

HIV: Human immunodeficiency virus; HCW: Health care worker; TB: tuberculosis.

## Competing interest statement

The authors declare that they have no competing interests.

## Authors' contributions

TH, KW, and AM contributed to the conception and design of the study. AF, JL, and AM lead the acquisition of data. AF, JL, TH, KW, and MvdW actively participated in the analysis and interpretation of data. AF, JL, and TH wrote the first draft of the manuscript, and KW, AM, and MvdW provided critical revision of manuscript. All authors read and approved the final manuscript.

## Pre-publication history

The pre-publication history for this paper can be accessed here:

http://www.biomedcentral.com/1471-2458/12/56/prepub

## References

[B1] WHO Global Tuberculosis Control: Surveillance, Planning, Financing. WHO Report 2004 2004 Geneva, Switzerland ISBN 92 4 156264 1

[B2] Statistics South Africa Mortality and causes of death in South Africa, 1997-2003; Findings from death notification (Statistical Release P0309.3) 2005 http://www.statssa.gov.za Accessed on 15 May 2011

[B3] Pablos-Mendez A Knirsch CA Barr RG Lerner BH Frieden TR Nonadherence in tuberculosis treatment: predictors and consequences in New York City Am J Med 1997 102 2 164 170 10.1016/S0002-9343(96)00402-0 9217566

[B4] Connolly C Davies GR Wilkinson D Who fails to complete tuberculosis treatment? Temporal trends and risk factors for treatment interruption in a community-based directly observed therapy programme in a rural district of South Africa Int J Tuberc Lung Dis 1999 3 12 1081 1087 10599011

[B5] Kharsany AB Connolly C Olowolagba A Abdool Karim SS Abdool Karim Q TB treatment outcomes following directly-observed treatment at an urban outpatient specialist TB facility in South Africa Trop Doct 2006 36 1 23 25 10.1258/004947506775598770 16483424

[B6] Holtz TH Lancaster J Laserson KF Wells CD Thorpe L Weyer K Risk factors associated with default from multidrug-resistant tuberculosis treatment, South Africa, 1999-2001 Int J Tuberc Lung Dis 2006 10 6 649 655 16776452

[B7] Castelnuovo B A review of compliance to anti tuberculosis treatment and risk factors for defaulting treatment in Sub Saharan Africa Afr Health Sci 2010 10 4 320 324 21416032 PMC3052808

[B8] Santha T Garg R Frieden TR Chandrasekaran V Subramani R Gopi PG Selvakumar N Ganapathy S Charles N Rajamma J Risk factors associated with default, failure and death among tuberculosis patients treated in a DOTS programme in Tiruvallur District, South India, 2000 Int J Tuberc Lung Dis 2002 6 9 780 788 12234133

[B9] Jaggarajamma K Sudha G Chandrasekaran V Nirupa C Thomas A Santha T Muniyandi M Narayanan PR Reasons for non-compliance among patients treated under Revised National Tuberculosis Control Programme (RNTCP), Tiruvallur district, south India Indian J Tuberc 2007 54 3 130 135 17886701

[B10] Chandrasekaran V Gopi PG Santha T Subramani R Narayanan PR Status of re-registered patients for tuberculosis treatment under DOTS programme Indian J Tuberc 2007 54 1 12 16 17455418

[B11] WHO Adherence to long-term therapies: evidence for action 2003 Geneva: World Health Organization

[B12] Lauristen JM Bruus M A comprehensive tool for validated entry and documentation of data EpiData Entry 2006 v.3.1 Odense, Denmark: The EpiData Association

[B13] StataCorp Statistical Softward: Release 9.0 2008 College Station, TX: Stata Corporation

[B14] Hane F Thiam S Fall AS Vidal L Diop AH Ndir M Lienhardt C Identifying barriers to effective tuberculosis control in Senegal: an anthropological approach Int J Tuberc Lung Dis 2007 11 5 539 543 17439678

[B15] Mishra P Hansen EH Sabroe S Kafle KK Adherence is associated with the quality of professional-patient interaction in Directly Observed Treatment Short-course, DOTS Patient Educ Couns 2006 63 1-2 29 37 10.1016/j.pec.2005.08.006 16242297

[B16] Vijay S Kumar P Chauhan LS Vollepore BH Kizhakkethil UP Rao SG Risk factors associated with default among new smear positive TB patients treated under DOTS in India PLoS ONE 2010 5 4 e10043 10.1371/journal.pone.0010043 20386611 PMC2850369

[B17] Comolet TM Rakotomalala R Rajaonarioa H Factors determining compliance with tuberculosis treatment in an urban environment, Tamatave, Madagascar Int J Tuberc Lung Dis 1998 2 11 891 897 9848609

[B18] Munro S Lewin S Swart T Volmink J A review of health behaviour theories: how useful are these for developing interventions to promote long-term medication adherence for TB and HIV/AIDS? BMC Public Health 2007 7 104 10.1186/1471-2458-7-104 17561997 PMC1925084

[B19] Driver CR Matus SP Bayuga S Winters AI Munsiff SS Factors associated with tuberculosis treatment interruption in New York City J Public Health Manag Pract 2005 11 4 361 368 15958938 10.1097/00124784-200507000-00017

[B20] Kapella BK Anuwatnonthakate A Komsakorn S Moolphate S Charusuntonsri P Limsomboon P Wattanaamornkiat W Nateniyom S Varma JK Directly observed treatment is associated with reduced default among foreign tuberculosis patients in Thailand Int J Tuberc Lung Dis 2009 13 2 232 237 19146753

[B21] Naidoo P Dick J Cooper D Exploring tuberculosis patients' adherence to treatment regimens and prevention programs at a public health site Qual Health Res 2009 19 1 55 70 18997153 10.1177/1049732308327893

[B22] Suri A Gan K Carpenter S Voices from the field: perspectives from community health workers on health care delivery in rural KwaZulu-Natal, South Africa J Infect Dis 2007 196 Suppl 3 S505 S511 18181702 10.1086/521122

[B23] Edginton ME Sekatane CS Goldstein SJ Patients' beliefs: do they affect tuberculosis control? A study in a rural district of South Africa Int J Tuberc Lung Dis 2002 6 12 1075 1082 12546115

[B24] Rowe KA Makhubele B Hargreaves JR Porter JD Hausler HP Pronyk PM Adherence to TB preventive therapy for HIV-positive patients in rural South Africa: implications for antiretroviral delivery in resource-poor settings? Int J Tuberc Lung Dis 2005 9 3 263 269 15786888

[B25] Garner P Smith H Munro S Volmink J Promoting adherence to tuberculosis treatment Bull World Health Organ 2007 85 5 404 406 10.2471/BLT.06.035568 17639229 PMC2636642

[B26] Getahun H Maher D Contribution of 'TB clubs' to tuberculosis control in a rural district in Ethiopia Int J Tuberc Lung Dis 2000 4 2 174 178 10694097

[B27] Jakubowiak WM Bogorodskaya EM Borisov SE Danilova ID Kourbatova EV Risk factors associated with default among new pulmonary TB patients and social support in six Russian regions Int J Tuberc Lung Dis 2007 11 1 46 53 17217129

[B28] Hasker E Khodjikhanov M Usarova S Asamidinov U Yuldashova U van der Werf MJ Uzakova G Veen J Default from tuberculosis treatment in Tashkent, Uzbekistan; who are these defaulters and why do they default? BMC Infect Dis 2008 8 97 10.1186/1471-2334-8-97 18647400 PMC2492865

[B29] Gelmanova IY Keshavjee S Golubchikova VT Berezina VI Strelis AK Yanova GV Atwood S Murray M Barriers to successful tuberculosis treatment in Tomsk, Russian Federation: non-adherence, default and the acquisition of multidrug resistance Bull World Health Organ 2007 85 9 703 711 10.2471/BLT.06.038331 18026627 PMC2636414

[B30] Bhagat VM Gattani PL Factors affecting tuberculosis retreatment defaults in Nanded, India Southeast Asian J Trop Med Public Health 2010 41 5 1153 1157 21073036

[B31] Kaner EF Beyer F Dickinson HO Pienaar E Campbell F Schlesinger C Heather N Saunders J Burnand B Effectiveness of brief alcohol interventions in primary care populations Cochrane Database Syst Rev 2007 2 CD004148 10.1002/14651858.CD004148.pub3 17443541

[B32] Whitlock EP Polen MR Green CA Orleans T Klein J Behavioral counseling interventions in primary care to reduce risky/harmful alcohol use by adults: a summary of the evidence for the U.S. Preventive Services Task Force Ann Intern Med 2004 140 7 557 568 15068985 10.7326/0003-4819-140-7-200404060-00017

[B33] Banerjee A Harries AD Nyirenda T Salaniponi FM Local perceptions of tuberculosis in a rural district in Malawi Int J Tuberc Lung Dis 2000 4 11 1047 1051 11092717

[B34] Brouwer JA Boeree MJ Kager P Varkevisser CM Harries AD Traditional healers and pulmonary tuberculosis in Malawi Int J Tuberc Lung Dis 1998 2 3 231 234 9526196

[B35] Barker RD Millard FJ Malatsi J Mkoana L Ngoatwana T Agarawal S de Valliere S Traditional healers, treatment delay, performance status and death from TB in rural South Africa Int J Tuberc Lung Dis 2006 10 6 670 675 16776455

[B36] Harper ME Hill PC Bah AH Manneh K McAdam KP Lienhardt C Traditional healers participate in tuberculosis control in The Gambia Int J Tuberc Lung Dis 2004 8 10 1266 1268 15527161

[B37] Colvin M Gumede L Grimwade K Maher D Wilkinson D Contribution of traditional healers to a rural tuberculosis control programme in Hlabisa, South Africa Int J Tuberc Lung Dis 2003 7 9 Suppl 1 S86 S91 12971659

[B38] Pinidiyapathirage J Senaratne W Wickremasinghe R Prevalence and predictors of default with tuberculosis treatment in Sri Lanka Southeast Asian J Trop Med Public Health 2008 39 6 1076 1082 19062698

[B39] Chang KC Leung CC Tam CM Risk factors for defaulting from anti-tuberculosis treatment under directly observed treatment in Hong Kong Int J Tuberc Lung Dis 2004 8 12 1492 1498 15636497

[B40] Chan-Yeung M Noertjojo K Leung CC Chan SL Tam CM Prevalence and predictors of default from tuberculosis treatment in Hong Kong Hong Kong Med J 2003 9 4 263 268 12904614

[B41] Jha UM Satyanarayana S Dewan PK Chadha S Wares F Sahu S Gupta D Chauhan LS Risk factors for treatment default among re-treatment tuberculosis patients in India, 2006 PLoS ONE 2010 5 1 e8873 10.1371/journal.pone.0008873 20111727 PMC2810342

[B42] Weyer K Case study: South Africa Bull World Health Organ 2007 85 5 391 10.2471/BLT.06.036004

[B43] Ntshanga SP Rustomjee R Mabaso ML Evaluation of directly observed therapy for tuberculosis in KwaZulu-Natal, South Africa Trans R Soc Trop Med Hyg 2009 103 6 571 574 10.1016/j.trstmh.2009.03.021 19394664

[B44] Clarke M Dick J Zwarenstein M Lombard CJ Diwan VK Lay health worker intervention with choice of DOT superior to standard TB care for farm dwellers in South Africa: a cluster randomised control trial Int J Tuberc Lung Dis 2005 9 6 673 679 15971396

[B45] Dick J Lombard C Shared vision-a health education project designed to enhance adherence to anti-tuberculosis treatment Int J Tuberc Lung Dis 1997 1 2 181 186 9441085

[B46] Thiam S LeFevre AM Hane F Ndiaye A Ba F Fielding KL Ndir M Lienhardt C Effectiveness of a strategy to improve adherence to tuberculosis treatment in a resource-poor setting: a cluster randomized controlled trial JAMA 2007 297 4 380 386 10.1001/jama.297.4.380 17244834

[B47] Gandhi NR Moll AP Lalloo U Pawinski R Zeller K Moodley P Meyer E Friedland G Successful integration of tuberculosis and HIV treatment in rural South Africa: the Sizonq'oba study J Acquir Immune Defic Syndr 2009 50 1 37 43 10.1097/QAI.0b013e31818ce6c4 19295333

[B48] Vree M Huong NT Duong BD Sy DN Van le N Co NV Cobelens FG Borgdorff MW Mortality and failure among tuberculosis patients who did not complete treatment in Vietnam: a cohort study BMC Public Health 2007 7 134 10.1186/1471-2458-7-134 17605770 PMC1925078

[B49] Botha E Den Boon S Verver S Dunbar R Lawrence KA Bosman M Enarson DA Toms I Beyers N Initial default from tuberculosis treatment: how often does it happen and what are the reasons? Int J Tuberc Lung Dis 2008 12 7 820 823 18544210

[B50] Korenromp EL Bierrenbach AL Williams BG Dye C The measurement and estimation of tuberculosis mortality Int J Tuberc Lung Dis 2009 13 3 283 303 19275787

[B51] Hopewell PC Pai M Maher D Uplekar M Raviglione MC International standards for tuberculosis care Lancet Infect Dis 2006 6 11 710 725 10.1016/S1473-3099(06)70628-4 17067920

[B52] Bateman ED Feldman C Mash R Fairall LR English RG Jithoo A Systems for the management of respiratory disease in primary care - an international series: South Africa Prim Care Respir J 2009 18 2 69 75 19173089 10.3132/pcrj.2009.00009 PMC6619249

